# Alcohol Abuse in Pregnant Women: Effects on the Fetus and Newborn, Mode of Action and Maternal Treatment

**DOI:** 10.3390/ijerph7020364

**Published:** 2010-01-27

**Authors:** Asher Ornoy, Zivanit Ergaz

**Affiliations:** 1 Laboratory of Teratology, The Institute of Medical Research Israel Canada, Hadassah Medical School and Hospital, The Hebrew University of Jerusalem, Ein Kerem, P.O. Box 12271, Jerusalem, 91120, Israel; E-Mail: zivanit@hadassah.org.il; 2 Department of Neonatology, Hadassah Medical School and Hospital, Hadassah Medical Center, Hebrew University, P.O. Box 24035, Jerusalem, 91240, Israel

**Keywords:** ethanol, pregnancy outcome, fetal alcohol syndrome

## Abstract

Offspring of mothers using ethanol during pregnancy are known to suffer from developmental delays and/or a variety of behavioral changes. Ethanol, may affect the developing fetus in a dose dependent manner. With very high repetitive doses there is a 6–10% chance of the fetus developing the fetal alcoholic syndrome manifested by prenatal and postnatal growth deficiency, specific craniofacial dysmorphic features, mental retardation, behavioral changes and a variety of major anomalies. With lower repetitive doses there is a risk of “alcoholic effects” mainly manifested by slight intellectual impairment, growth disturbances and behavioral changes. Binge drinking may impose some danger of slight intellectual deficiency. It is advised to offer maternal abstinence programs prior to pregnancy, but they may also be initiated during pregnancy with accompanying close medical care. The long term intellectual outcome of children born to ethanol dependent mothers is influenced to a large extent by the environment in which the exposed child is raised.

## 1. Introduction

Several drugs and chemicals are known to be teratogenic to the human embryo when administered during pregnancy, especially during the period of organogenesis. The evidence for their teratogenicity has been shown by human epidemiologic and clinical studies, as well as in studies carried out in animals such as rats, mice, rabbits and primates. These teratogenic insults occurring during embryonic life may be present immediately after birth, at infancy or even later in life, especially if the damage involves the Central Nervous System (CNS) [1]. Moreover, many of the insults to the CNS may occur in the second and third trimesters of pregnancy, when most other organs have already passed the stage of active organogenesis. Briefly, the main stages of the human CNS development are the formation of the neural folds, their closure to form the neural tube that closes completely towards the end of the fourth week post fertilization, the formation of the main brain vesicles during weeks 5 and 6 and the tissue surrounding the central canal that develops to the different brain vesicles. However, the cortical plate starts to develop mainly during weeks 8–9 post fertilization, and the cerebellar cortex is developing later, mainly during the second and third trimesters of pregnancy. The cerebral cortex continues to develop actively throughout gestation and even in the early postnatal life, mainly by forming the different cortical layers, neuronal growth and sprouting, synapse formation and myelinization. It is therefore expected that psychotropic agents such as ethanol are able to affect the development of the CNS almost throughout the entire pregnancy [1,2]. Hence, such late effect will not necessarily be manifested by morphological changes in the CNS but rather by more subtle changes in the intellectual capacity, learning ability, attention span and behavior.

In this review we will discuss the possible effects of ethanol use during pregnancy on the human embryo and fetus. We will survey studies concerning ethanol-abusing women either throughout pregnancy or following sporadic use. We will also discuss some animal studies especially those related to the mechanisms of action of ethanol. Unlike most drugs that impact the CNS or other organs, ethanol may affect both the mother and embryo, inducing mainly, but not exclusively, behavioral problems and intellectual deficits.

## 2. History of Alcohol Effects in Pregnancy

The history of maternal alcoholism and its effect on development of the offspring goes back to the Bible and to the early Greek mythology. Samuel the prophet forbids Samson’s mother from drinking wine during her pregnancy because she is going to give birth to a child blessed by God with special power, and the bridal couple, in Carthage, was forbidden to drink wine in the wedding night to prevent a birth of a defective child. In 1834 a report to the House of Commons indicated that some of the alcoholic mothers gave birth to “a starved, shrivelled and imperfect look” infants and in 1900 Sullivan reported an increase in the rate of abortions and stillbirth as well as increased frequency of epilepsy among live-born infants of chronic alcohol abusing women [3]. The teratogenic effects of ethanol on human fetuses were first reported by Lemoine *et al.* in 1968, describing a common pattern of birth defects in 127 children born to alcoholic mothers in France. This included growth deficiency, psychomotor retardation, low IQ, and atypical EEG [4]. Alcohol was used at the time to prevent premature labor. However, in these women alcohol was given in rather late phases of pregnancy, post the organogenetic period and therefore resulted in no morphologic changes and apparently only very little (if at all) effects on behavior. Although the adverse/harmful effects of alcohol use during pregnancy have been suggested for decades, it was rather difficult to formally document or diagnose the constellation of problems observed in these children, not until guidelines for fetal alcohol syndrome (FAS) were established [5].

## 3. Genetic factors

### 3.1. Genetic Susceptibility to Alcoholism

Increased levels of acetaldehyde, the toxic intermediary product of alcohol metabolism may result from polymorphism of the loci encoding alcohol dehydrogenase (ADH) and or acetaldehyde dehydrogenase (ALDH2) [6]. Several studies reported high polymorphism (*) among alcohol abusing persons. Associations between high ADH3*2 and low ADH 2*2 and ALDH*2 alleles were found frequently among alcohol dependent men in Mexico [7]. Both ALDH2*1/*2 and ALDH2*2/*2 were lower and ADH2*1/*1 and ALDH2*1/*1 higher among alcoholic Korean men [8]. The ADH2*1 genotype was increased among alcoholics and the coexistence of ADH2*1/*1 and ALDH2*1/*1 was 6.4−9.6 enhanced among Japanese alcoholics compared to controls [9]. Mexican-Americans were found to have high frequency of the allele CYP2E1c2 which is associated with early onset alcoholism [10].

### 3.2. Genetic Susceptibility to Fetal Alcohol Effect

Discordance in the development of fetal alcohol syndrome was found among dizygotic twins, compared to concordance in monozygotic twins [11]. A sevenfold increase in the risk of FAS was found among African-Americans compared to Caucasians [10]. In a computational study the MAPK (Mitogen activated protein Kinase), TGF-β and the Hedgehog signaling pathways were found associated with deleterious effects of alcohol in experimental animal studies [12]. A protective effect of the allele ADH1B was found in various studies. ADH1B*2 among mixed ancestry[13] south African population and ADH1B*3 in African Americans [14]. Maternal CYP17 A1A1 genotype was associated with fetal growth restriction among alcoholic women in England [15]. Paternal drinking was also offered as a risk factor for FAS. It has been documented that alcohol can reduce the levels and activity of DNA methyltransferases resulting in DNA hypomethylation and that reduced methyltransferase activity can cause activation of normally silenced genes sperm. It was therefore hypothesized that these epigenetic changes in imprinted genes may be transmitted through fertilization and may alter the critical gene expression dosages required for normal prenatal development resulting in offspring with features of FAS. When DNA from male volunteers was bisulfite treated, a pattern of increased demethylation in two differentially methylated regions (DMRs), H19 and IG-DMR, correlated with their alcohol consumption levels [16].

### 3.3. Familial Susceptibility

Fetal alcohol exposure was a risk factor for alcohol, drug, and nicotine dependence among adoptees when evaluated between 18–45 years of age. Other variables including adoptive parents factors and peer influence were less statistically significant [17]. Increased risk of alcohol consumption among adolescents 14 years old was found in those whose mothers reported alcohol consumption before, during and after pregnancy. There was an increased risk of early alcohol initiation among the ones exposed to greater alcohol use during pregnancy [18]. In another study drinking rates at 21 years did not relate to maternal drinking during pregnancy after adjustment for demographics and other potential exposures. The negative consequences associated with prenatal alcohol exposure were that it tripled the odds that the offspring at age 21 will have alcohol dependence [19].

## 4. Effects on the Developing Embryo and Fetus

### 4.1. Fetal Alcohol Syndrome (FAS) and Alcohol Effects

Basically there seem to be three categories of prenatal exposure to ethanol related to the amount of alcohol ingested. Exposure to heavy drinking (over 48–60 gr. ethanol/day) that may cause fetal alcohol syndrome; exposure to moderately high drinking, between 24–48 gr. ethanol/day which may result mostly in “alcohol effects” (the differences between these categories are not sharp) and binge drinking occasions with intakes of 4–5 drinks of ethanol (altogether more than 90 gr. ethanol/drink) [20,21]. The amount of alcohol ingested the length of period using alcohol and the developmental stage of the embryo and fetus at exposure mediate the effects of ethanol intake on the developing fetus. Alcohol drinking, even in moderate amounts, is also associated with an increased risk of spontaneous abortions, especially in the first trimester of pregnancy and with infertility in males and females [20]. It is, however, important to note that a meta-analysis of reports on the incidence of fetal malformations in moderately alcohol abusing women during pregnancy did not show an increase in the rate of congenital anomalies [22].

Alcohol drinking was associated with increased risk of fetal death. Hazard ratio was 1.55 among women in the Danish National Birth Cohort who reported binge drinking three or more times during pregnancy [23]. An odds ratio of 1.4 for stillbirth was also found in a cohort of 3,508 singleton pregnancies in Missouri, which increased to 1.7 in women consuming five or more drinks per week [24].

It has been demonstrated repeatedly that high alcohol consumption during pregnancy may seriously affect the developing embryo. The severity of the malformations ranges from FAS, which is evident in 4–6% of infants of heavy drinking mothers, to minor effects, such as low birth weight, Intra Uterine Growth Retardation (IUGR), a slight reduction in IQ of the infants and increased rate of congenital anomalies [3,5,25,26]. Intra uterine growth retardation as well as postnatal long-term height and weight deficits is well demonstrated among children born to ethanol using women. Further, Covington *et al.* found a moderating effect of the maternal age on the children’s weight at age 7, as children born to women over 30 years of age at the time of birth had significantly lower weight as compared to those born to younger women [27].

The most common, serious and specific syndrome of alcohol effects in pregnancy—FAS—has been described only for regular/daily high dose alcohol users [3,5,28]. Recognition of the syndrome was made by Dr. David Smith and Dr. Kenneth Jones in 1973 based on the evaluation of eight children born to mothers who were defined as chronic alcoholic [29]. The principal features of FAS were determined as prenatal and postnatal growth deficiency, short stature, developmental delay, microcephaly, fine-motor dysfunction and facial dysmorphism. In addition there may be cleft palate, joint and cardiac anomalies and, altered palmar creases. The above described facial dysmorphism tends to improve with the advancement in age of the affected individuals.

### 4.2. Criteria for Diagnosis

The criteria are based on growth deficiency, facial phenotype, central nervous system damage and evidence of intrauterine alcohol exposure. The more commonly used scores are the 1996 institute of medicine criteria [30] and the Washington criteria [31]. Hoyme *et al.* offered revised criteria applicable for pediatric practice by scoring the clinical findings: height <10%; weight <0%; occipito-frontal circumference <10%; inner canthal distance <10%; palpebral fissure length <10%; attention-deficit/hyperactivity disorder; fine motor dysfunction; mid-facial hypoplasia; “railroad track” ears; strabismus; ptosis; nonracial epicanthal folds; flat nasal bridge; anteverted nares; long philtrum; smooth philtrum; thin vermilion border of upper lip; prognathism; cardiac murmur; cardiac malformations; hypoplastic nails; decreased pronation/supination of elbow; clinodactyly of fifth fingers; camptodactyly; “hockey stick” palmar creases and hirsutism [32]. Even several of these clinical findings are sufficient for diagnosis if there is a positive history of alcohol exposure.

## 5. Anomalies of the Organ Systems

Alcohol is known to affect not only the CNS but also organs that are developmentally related to CNS derivatives, including those developmentally dependent on neural crest cells like the cranio-facial complex and the heart.

### 5.1. Oro-Facial Clefts

A number of reports addressed a potential correlation between alcohol consumption and oral clefts. In a case control surveillance study Meyer *et al.* [33] collected 5,956 liveborn infants with: Cleft Palate (CP), Cleft Lip (CL), or both CP and CL. Based on the maternal report of alcohol use during the first 4 month of pregnancy, the authors failed to link low levels of alcohol use and oral clefts. Even the highest level of alcohol consumption did not result in a higher number of infants born with a cleft than with the use of less than one drink per week or less than one drink per drinking day. In addition, folic-acid supplemented multivitamins used by some of the women did not modify the association between oral clefts and ethanol consumption. In contrast, Romitti *et al.* [34] based on the data from the National Birth Defects Prevention Study, found a weak correlation between average periconceptional alcohol consumption and all oro-facial clefts (combined and isolated clefts). A moderate link was identified for multiple clefts and for Pierre-Robin syndrome, although based on small numbers. An increased risk of oro-facial clefts was also observed among infants born to binge-drinking (five or more drinks per occasion) mothers exposed in the first trimester of pregnancy. Maternal binge-drinking may be particularly harmful since it results in a greater peak of blood ethanol concentrations [35]. In one study the mutated ADH1C allele gene (that is involved in ethanol metabolic pathways) among carried children was found protective against the risk of oral clefts with the maternal genotype playing a less important role than the child’s [36].

### 5.2. Cardiac Anomalies

It is accepted that about one third of children with alcohol embryopathy will also have congenital cardiac problems. A higher risk of VSD [37], ASD [38] D-transposition [39], conotruncal heart defect [40], coarctation and hypoplastic aortic arch [41] were associated with maternal alcohol consumption. Krasemann and Klingebiel [42] retrospectively reviewed data of electrocardiogram (ECG) and echocardiogram (EchoCG) of all patients with clinical signs of alcoholic embryopathy between the years 1976 and 2003. Measurements of ECG and EchoCG often showed shortened QT in about half and shortened left ventricular diameter in about a quarter of the patients with alcoholic embryopathy consequently concluding that alcohol abuse during pregnancy as a primary toxin can lead to minor cardiac abnormalities, even without structural congenital cardiac defects.

### 5.3. Neural Tube Defects

Maternal alcohol consumption early in pregnancy was found to be related to increased risk of neural tube defects. Chen found in the literature nine cases of NTD related to prenatal exposure to ethanol, but speculated that this effect may result from the folate deficiency induced by alcohol abuse [43]. A hazard ratio of 1.6 and 2.1 for neural tube defect was found in women who consumed ethanol during the periconceptual period, less than once and more than once a week respectively [39]. Opposed to these findings, periconceptual maternal alcohol use did not reveal increased risk for neural tube defects using a population based case control study between 1989–1991 in California [44].

### 5.4. Renal Anomalies

Binge drinking during the second month of pregnancy was associated with bilateral renal agenesis or hypoplasia among 75 infants evaluated between 1997–2003 in North Carolina [45].

### 5.5. Atopic Dermatitis

Alcohol consumption during pregnancy was associated with increased risk of atopic dermatitis in early infancy that resolved during childhood. This effect was mainly increased when the two parents had allergic disease. The highest risk was seen in high-risk infants of mothers who consumed four or more drinks per week at 30 weeks of gestation [46].

### 5.6. Behavioral and Developmental Changes

Alcohol is considered one of the risk factors for Attention Deficit Hyperactivity Disorder (ADHD), independently of prenatal nicotine exposure or other familial risk factor. A positive correlation between alcohol and ADHD was reported in 26 prenatally alcohol exposed children. Of the 24 children followed up, 10 were diagnosed as having ADHD, 2 were with Asperger syndrome (a relatively mild form of autistic spectrum disorder) and one with mild mental retardation. The severity of the disorder correlated in a linear pattern with the amount of alcohol used by the mother during pregnancy. Discontinuation of the alcohol consumption by the 12th week resulted in normally developed children, demonstrating the fact that the cerebral cortex is more vulnerable to these effects of ethanol from the second trimester of pregnancy, post the organogenetic period. Moreover, consumption of less than one alcoholic drink per day in the last three months of pregnancy, in spite of heavier drinking earlier, did not result in ADHD, learning disabilities or cognitive impairment at the age of 14 years [47].

Among 10 alcohol dependent adults who had maternal and paternal history of alcoholism, 8 had ADHD; seven of them were at risk of suicide, and all had a history of violence during intoxication. When compared to a group of 185 matched control alcohol addicts without a family history of alcoholism, none of the controls had ADHD, one had a risk of suicide and two were violent when drunk [48]. It has been difficult to define and characterize developmental risks associated with binge drinking or moderate drinking in pregnancy [49] and some studies failed to demonstrate an association between alcohol exposure and sustained attention performance in school age children [50].

Alcohol in pregnancy may affect intellectual ability that together with attention span and behavior are being considered higher functions of the cerebral cortex. Studies in 7 year-old school children following prenatal exposure to moderate amount of alcohol showed a decrement of 7 points in IQ [51]. Nine years old participants with FAS were significantly weaker than control children in reading, spelling, addition, subtraction, phonological awareness, and other tests of early literacy. A deficit in phonological awareness, a key pre-function for reading may be a primary cognitive phenotypic characteristic in children with FAS. Syllable manipulation, letter sound knowledge, written letters, word reading and non-word reading and spelling were improved after nine months manipulation class [52].

Delayed motor functions, mostly fine motor skills were found in 22–68 months old FAS children [53]. In addition, alcohol may also affect the cerebellum. In the human cerebellum, Purkinje cell migration is completed and their dendritic outgrowth begins around gestational week 26 extending to the third trimester of pregnancy. Consequently, a period of enhanced vulnerability of Purkinje cells to binge alcohol exposure in humans would be predicted near the end of the second trimester and may extend over the third trimester [54]. Cerebellar developmental disorders and disproportionate reduction in the anterior cerebellar vermis have been identified by MRI in children that were exposed prenatally to alcohol during each trimester of pregnancy [55]. Children with FAS were found to have the frontal lobes smaller with lower choline concentrations in MRS (MR spectroscopy) as a marker of cell membrane stabilizer and myelinization. The caudate nucleus was found disproportionately smaller in children with neuropsychological impairment. The prevalence to have one or more brain regions 2 or more SD below mean size in controls was proportional to their neurological damage [56]. Decreased cerebellar growth and decreased cranial to body growth in fetuses of alcohol abusing mothers was also observed on fetal ultrasound performed on the 18 week of gestation [57]. If the mothers stopped drinking at the beginning of pregnancy, cerebellar growth was normal.

### 5.7. Psychiatric Disorders

Adults exposed to binge drinking while in utero were found to have increased rate of somatoform disorders, substance dependence, paranoid, passive aggressive, anti social and other personality disorders [58].

## 6. Mechanism of Alcohol Teratogenicity

Different mechanisms have been offered to explain the teratogenic effects of alcohol on the developing embryo. They include the following: (1) Increased oxidative stress; (2) Disturbed glucose, protein, lipid and DNA metabolism; (3) Impaired neurogenesis and increased cellular apoptosis, especially of neural crest cells [26,59–61]; 4) Endocrine effect; 5) Effects on gene expression.

### 6.1. Oxidative Stress

Alterations in the redox status in the CNS was supported by studies demonstrating ethanol—mediated changes in the production and/or activity of endogenous antioxidants in various organs, including the cerebellum and placenta [26,60,61].

Ethanol can induce oxidative stress directly by formation of free radicals which react with different cellular compounds, or indirectly by reducing intracellular antioxidant capacity, such as decreased glutathione peroxidase levels. A significant increase in oxidative stress was demonstrated in placental villous tissue following two hours of ethanol perfusion, primarily involving the nitric oxide pathway in the trophoblast and DNA damage in the villous stromal cells [61]. Alcohol-induced oxidative stress was also found to increase lipid peroxidation and damage protein and DNA. However, there is lack of data from human clinical studies. No significant different in urine 2,3-dinor-6-keto-prostaglandin F1α, or 11-dehydro-thromboxane B2 8-isoprostane F2α an oxidative stress markers was found between pregnant women who drank heavily and those who abstained [62].

### 6.2. Disturbed Prostaglandin Synthesis

Alcohol is known to affect prostaglandins, hence, influencing fetal development and parturition. When mice were treated with aspirin (a prostaglandin synthesis inhibitor) prior to alcohol exposure, the alcohol-induced malformations were reduced by 50% in comparison to mice treated with aspirin after alcohol exposure [63]. Urinary 6-keto-PGF1 alpha and 2,3-dinor-6 keto PG F1 alpha were higher in heavily drinking mothers and infants who suffered from FAS compare to abstinent mothers and infants. High levels of thromboxane B2 in urine were also found in the infants of the drinking mothers but without correlation to FAS [64].

### 6.3. Effects on Neurons

Several studies in rats and mice have shown that in utero exposure to alcohol caused structural defects in the hippocampus, cerebellum and neural crest cells with increased cell death [59,60,65].

### 6.4. Endocrine Effect

An effect of prenatal alcohol exposure on the limbic, hypothalamic pituitary−adrenal axis was shown when higher cortisol and heart rate levels were found in 5–7 month-old infants during emotional stress. The effect differed among the genders, with boys having higher cortisol levels and girls higher heart rate [66].

### 6.5. Gene Expression

Neural progenitor cells that were isolated from normal second trimester fetal human brains and cultured for up to 72 hours in mitogenic media containing ethanol generated in these conditions neurosphere which diameter correlated positively with the increasing ethanol concentrations. Real-time PCR analysis showed that ethanol significantly altered the expression of genes involved in cell adhesion. There was an increase in the expression of alpha and beta Laminins 1, beta Integrins 3 and 5, Secreted phosphoprotein 1 and Sarcoglycan epsilon. Those changes may underlie aspects of neuro-developmental abnormalities in FAS [67]. In light of those different mechanisms of action, it is reasonable to presume that alcohol- induced teratogenicity is probably the result of injuries caused by several mechanisms [68].

## 7. Prevention and Treatment

### 7.1. Prevention

Unfortunately, there are only few reports demonstrating success in reducing drinking of alcohol in pregnancy, and these reports even declined from 1995–1999. The rate of binge-drinking and of chronic heavy drinking remained unchanged, suggesting that the education programs were not effective. Preventing alcohol abuse must, therefore, start with educational programs in schools. Preventing programs need to be primarily addressed towards high risk individuals and groups. Lately a motivational intervention to reduce the risk of an alcohol-exposed pregnancy in preconceptional women by information plus a brief motivational intervention or information resulted in twofold reduced risk of fetal alcohol exposure across the follow-up period [69].

### 7.2. Treatment during Pregnancy

Assuming that oxidative stress is one of the major routes of ethanol-induced damage, it is reasonable to supplement antioxidants, in effort to attenuate this damage. Antioxidants, such as Vitamin C, Vitamin E, folic acid, beta-caroten and flavonoids can be supplemented by food, therefore reversing other nutritional deficits common among this population [68]. However, to our knowledge, only few such programs exist, if at all.

### 7.3. Lactation

Alcohol is transferred to human milk, reaching levels similar to those in maternal serum, hence, women with heavy drinking should refrain from nursing. However, according to the American Academy of Pediatrics lactation is allowed, apparently depending on the daily dose of alcohol. The known side effects includes: drowsiness, diaphoresis, deep sleep, weakness, decrease in linear growth, abnormal weight gain and decreased milk ejection reflex with high maternal injestion [70]. If nursing mothers drink only small to moderate amounts of alcohol, they should wait 2–3 hours before nursing their infants.

### 7.4. Early Recognition of Fetal Alcohol Exposure

Early detection of neonates who had intrauterine alcohol exposure may enable effective social and educational intervention programs. The meconium fatty acid ethyl esters (FAEE), ethyl palmitate (E16:0), ethyl palmitoleate (E16:1), ethyl stearate (E18:0), ethyl oleate (E18:1), ethyl linoleate (E18:2), ethyl linolenate (E18:3), and ethyl arachidonate (E20:4) products of nonoxidative ethanol metabolism, have been established as a biomarker of fetal ethanol exposure. Among 682 meconium specimens anonymously collected in Canada FAEE analysis detected fivefold more ethanol-exposed pregnancies than reported by standard postpartum questionnaires in this population [71].

## 8. Prevention and Treatment of Alcohol Exposed Pregnant Animals

There seem to be no human studies trying to prevent the teratogenic effects of alcohol during pregnancy. However, there are several animal studies which demonstrated that antioxidants, as well as other agents, may be effective in reducing ethanol – induced embryotoxicity. Alcohol exposed C57BL/6J mice were injected twice with 2.9 g/kg, four hours apart of EUK-134 (a potent synthetic superoxide dismutase plus catalase mimetic) on their 9th day of pregnancy. EUK-134 supplementation induced a notable reduction in cell death of apical ectodermal ridge of the newly forming limb buds in ethanol exposed embryos and reduced the forelimb malformations by half (67.3% to 35.9%) [72].

Another support for the efficiency of antioxidants in attenuating the teratogenic effects of alcohol consumption throughout pregnancy comes from Wentzel *et al.* [73] who studied the effects of 5% vitamin E addition to food on the outcome of ethanol exposed rat pregnancies, showing a reduced rate of malformed or dead fetuses, but no change in the alcohol induced reduction of body weight.

Metadoxine accelerates ethanol metabolism by increase in acetaldehyde activity, ethanol and acetaldehyde plasma clearance and urinary elimination of ketones. A faster rate of ethanol elimination was found in patients with acute alcohol intoxication [74]. Reports on the safety of metadoxine in pregnant rats in therapeutic dosage were published in the Chinese literature [75] and may be a promising therapy for binge drinking during pregnancy.

## 9. Conclusions

Maternal alcohol ingestion in pregnancy may have deleterious effects on the CNS and other organs of the developing embryo and fetus, depending on the dose, duration and developmental stage of the embryo at exposure. These embryotoxic effects of alcohol were observed in many animal species. It is therefore important to reduce alcohol drinking during pregnancy to the minimum. However, as of today, it is still difficult to define the minimal dose that will affect the developing embryo and the exact dose response relationship. Educational interventions should start at school and in adolescents and young adults it should start before pregnancy. Early recognition of intrauterine alcohol exposed children may allow nutritional, behavioral and schooling support. Several studies on ethanol exposed pregnant animals are promising in regards with the possible amelioration of alcohol-induced embryotoxicity.
